# Capric acid secreted by *Saccharomyces boulardii* influences the susceptibility of *Candida albicans* to fluconazole and amphotericin B

**DOI:** 10.1038/s41598-021-86012-9

**Published:** 2021-03-22

**Authors:** Jakub Suchodolski, Daria Derkacz, Przemysław Bernat, Anna Krasowska

**Affiliations:** 1 Department of Biotransformation, Faculty of Biotechnology, University of Wroclaw, Joliot-Curie 14A, 50-383 Wrocław, Poland; 2 Department of Industrial Microbiology and Biotechnology, Faculty of Biology and Environmental Protection, University of Łódź, Banacha 12/16, 90-237 Lodz, Poland

**Keywords:** Antifungal agents, Fungi, Pathogens

## Abstract

The effect of capric acid, secreted by the probiotic yeasts *Saccharomyces boulardii*, was evaluated on the activities of fluconazole (FLC) and amphotericin B (AMB) against pathogenic *Candida albicans* fungus. The findings indicated that capric acid may be a promising additive for use in combination with FLC. A FLC-capric acid combination led to reduced efflux activity of multidrug resistance (MDR) transporter Cdr1p by causing it to relocalize from the plasma membrane (PM) to the interior of the cell. The above effect occurred due to inhibitory effect of FLC-capric acid combination of ergosterol biosynthesis. However, capric acid alone stimulated ergosterol production in *C. albicans*, which in turn generated cross resistance towards AMB and inhibited its action (PM permeabilization and cytoplasm leakage) against *C. albicans* cells. This concluded that AMB should not be administered among dietary supplements containing capric acid or *S. boulardii* cells.

## Introduction

*Candida albicans* is a resident of human microbiota present on the skin, in the gastrointestinal tract and in the genital mucosae^1^. Under immunosuppression, *C. albicans* causes opportunistic infections, which may result in sepsis and multiple organ failure^2,3^.

There is limited range of antifungal drug classes available to combat candidiasis. One such drug class includes azoles, which inhibit ergosterol biosynthesis by targeting cytochrome P-450 lanosterol 14α-demethylase (CYP51A1, Erg11p) encoded by the *ERG11* gene^4^. However, fungi increasingly develop resistance towards azoles, including the most commonly prescribed fluconazole (FLC)^5–7^. In the case of *C. albicans*, two major resistance mechanisms have been reported. These involve structural alterations to the target enzyme by overexpression or point mutations of the *ERG11* gene^8^, as well as overproduction of multidrug resistance (MDR) transporters, which remove azoles from the fungal cell^9^. One of the alternative treatment option involves the use of polyenes, such as amphotericin B (AMB). Polyenes bind to ergosterol in the fungal plasma membrane (PM), inducing permeabilization and cytoplasm leakage^10^. However, polyenes are toxic towards the mammalian cells and treatment is associated with a number of adverse effects among patients^11^. Resistance towards polyenes is not common among clinical *C. albicans* isolates and include downproduction of ergosterol^12^. However, laboratory-delivered polyene-resistant *C. albicans* strains overproduce ergosterol^13^.

Even with antifungal therapy, invasive candidiases results in a 50–70% patient mortality rate^14^. Therefore, the development of new therapies for fungal diseases remains a challenging priority for modern medicine. The trend towards green chemistry has motivated the quest for natural compounds as novel antimicrobials, such as fatty acids (FAs)^15^, which have been described to possess antiviral, antibacterial and antifungal properties^16^. With regards to fungi, FAs are active against molds, dermatophytes, phytopathogens and yeast-like fungal species^17^. Although FAs are not as effective as polyenes or azoles, it has been reported that pathogenic fungi are less likely to become resistant to FAs^17^.

FAs are metabolites derived from various sources, including probiotic microorganisms. One such organism, probiotic yeast *Saccharomyces boulardii*, is known to produce caproic (C6:0), caprylic (C8:0) and capric (C10:0) acids^18^, the latter of which has been reported to possess high activity against *C. albicans*^19^. The toxic effects of capric acid on *C. albicans* include shrinking of the cytoplasm in this fungus^19^, as well as inhibition of *C. albicans* virulence factors, such as adherence and the formation of hyphae^18^.

Another promising approach to combating antifungal drug resistance is the identification of compounds which synergistically enhance the activity of currently available drugs^12^. Simultaneously, the use of compounds (such as drugs or dietary supplements) that would antagonistically inhibit the activity of antifungal drugs or have undesirable interactions with them is avoided in clinical settings^20,21^. In this study we aimed to investigate the effect of capric acid on the antifungal properties of FLC and AMB against *C. albicans*. We observed that capric acid co-administered with FLC inhibits the activity of Cdr1 transporter, suggesting that this may be a promising synergistic agent in combination with azole drugs. Conversely, we observed that treatment with capric acid leads to overproduction of ergosterol in *C. albicans* cells, leading to cross resistance towards AMB.

## Results

### Capric acid has a synergistic effect in combination with fluconazole, but an antagonistic effect in combination with amphotericin B

**Figure 1:**
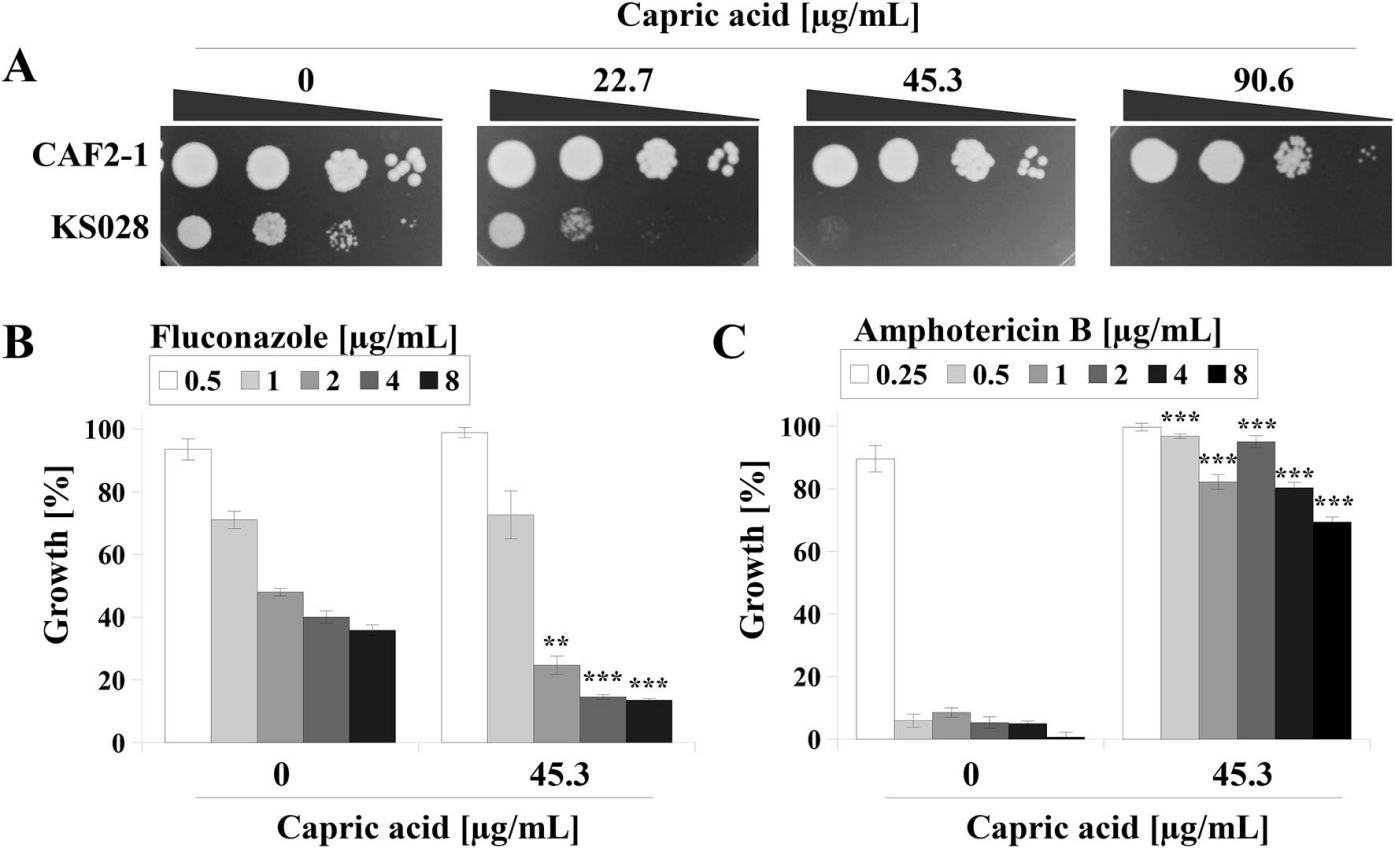
(**A**) Growth phenotypes of *C. albicans* CAF2-1 (parental strain) and KS028 (*erg11∆/∆*) strains after 48 h incubation at 28 °C in YPD medium with capric acid (0–90.6 µg/mL). (**B**) Percentage of growth of the *C. albicans* CAF2-1 strain in the presence of fluconazole (0.5–8 μg/mL, chart legend) and in presence of 45.3 μg/mL capric acid (means ± SD, *n* = 3). (**C**) Percentage of growth of the *C. albicans* CAF2-1 strain in the presence of amphotericin B (0.25–8 μg/mL, chart legend) and in presence of 45.3 μg/mL capric acid (means ± SD, *n* = 3). Statistical analyses were performed by comparing growth at the same fluconazole (**B**) or amphotericin B (**C**) concentrations between samples treated and untreated with capric acid. Statistical significance in all cases is presented as follows: **p < 0.01; ***p < 0.001.

The effect of capric acid treatment was evaluated against the in-house generated *C. albicans* KS028 strain, which features an *ERG11* gene deletion, depriving the cells of ergosterol (Fig. 1A). The *C. albicans* KS028 strain was reported to be unaffected by azoles or polyenes due to the absence of their respective targets Erg11p and ergosterol^22^. Thus, drug screening using the KS028 strain may allow the characterization of new synergistic or antagonistic drug combinations^23,24^.

The parental *C. albicans* strain (CAF2-1) continued to proliferate in the presence of all capric acid concentrations used in this study (Fig. 1A). Slight growth inhibition was observed upon treatment with 90.6 µg/mL capric acid. At a capric acid concentration of 22.7 µg/mL, a marked growth inhibition was observed in the KS028 strain. Complete growth inhibition of KS028 was observed upon treatment with 45.3 µg/mL capric acid. Based on these data, a 45.3 µg/mL capric acid concentration was selected for combination with FLC (Fig. 1B) or AMB (Fig. 1C).

Combination with capric acid enhanced the activity of FLC against *C. albicans* CAF2-1 (Fig. 1B). The growth of *C. albicans* at FLC concentrations of 2, 4 and 8 μg/mL was 50, 40 and 37%, respectively. Introduction of capric acid resulted in a reduction of CAF2-1 growth to 20, 17 and 16.5% in corresponding concentrations of FLC. In the case of AMB, it was observed that the presence of capric acid almost completely inhibited its activity against *C. albicans* CAF2-1 (Fig. 1C). Under control conditions, a complete reduction in *C. albicans* growth was observed at 0.25 µg/mL of AMB. The addition of capric acid resulted in 80–90% growth in the presence of a concentration range of 0.25–4 μg/mL AMB and 65% in the presence of a concentration of 8 μg/mL of AMB (Fig. 1C).

### Activity, localization, and expression of the Cdr1 transporter are altered in *C. albicans* treated with the capric acid or fluconazole–capric acid combination

**Figure 2:**
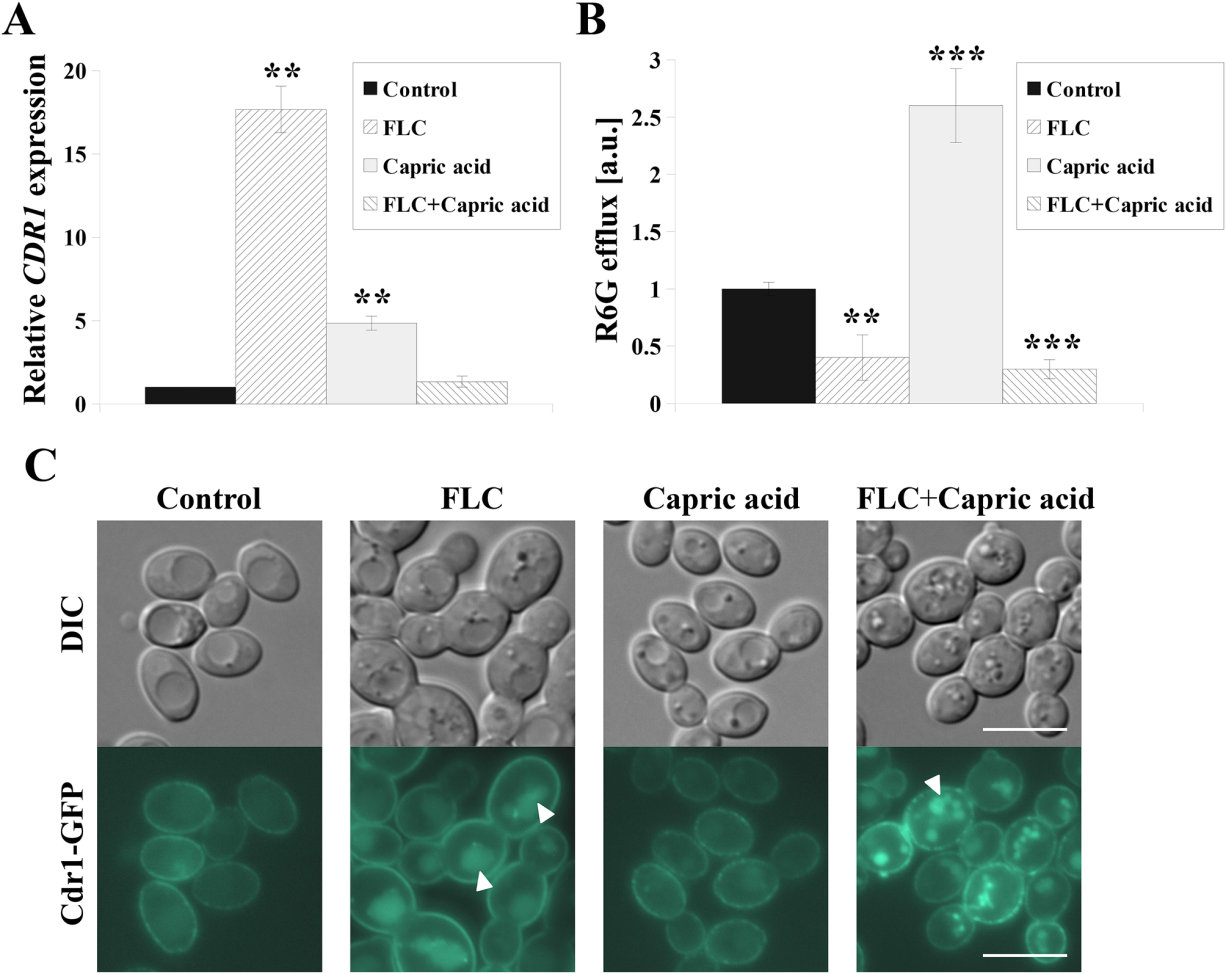
(**A**) Relative *CDR1* gene expression in the *C. albicans* CAF2-1. Gene expression levels are reported as means ± SD of 2^−ΔΔCT^ values (*n* = 3), normalized to 1 for control conditions. (**B**) Cdr1p-dependent rhodamine 6G (R6G efflux) in the *C. albicans* CAF2-1 normalized to = 1 for untreated control; means ± SD, *n* = 3. (**C**) Fluorescence micrographs of the subcellular localization of the Cdr1-GFP protein in the *C. albicans* KS052 (CAF2-1 *CDR1-GFP*). For the experiments *C. albicans* was grown for 8 h in the following conditions: control without antimicrobial agents, FLC—treated with fluconazole 4 μg/mL; Capric acid—treated with capric acid 45.3 μg/mL; FLC + Capric acid—simultaneously treated with fluconazole 4 μg/mL and capric acid 45.3 μg/mL. Statistical analysis was performed by comparing the *CDR1* expression level (**A**) or R6G fluorescence intensity (**B**) of cells treated with antimicrobial agent(s) with the corresponding untreated control. Scale bar = 5 μm. Statistical significance was presented as follows: **p < 0.01; ***p < 0.001.

Expression and activation of MDR transporters is the first line defense of *C. albicans* towards azole drugs^25^. Cdr1p belongs to the ATP-binding cassette family and is the most influential of the identified MDR transporters involved in FLC efflux in *C. albicans*^26^. The FLC–capric acid combination lead to greater growth inhibition in *C. albicans* than treatment with FLC alone (Fig. 1). Thus, we aimed to investigate the relative effect of capric acid and FLC-capric acid combination on the expression and activity of *CDR1*, as well as on the localization of Cdr1p (Fig. 2).

FLC alone induced increased *CDR1* expression almost 18-fold and capric acid alone almost fivefold compared with untreated *C. albicans* cells (Fig. 2A). However, an only 1.3-fold increase in *CDR1* expression was identified after treating *C. albicans* with combination of both compounds.

Cdr1p activity was determined by monitoring the extracellular fluorescence intensity of its fluorescent substrate, R6G^27^. Treatment with FLC and FLC-capric acid combination resulted in 60% and 70% lower efflux activity of Cdr1, respectively (Fig. 2B). On the other hand, treatment with capric acid alone lead to 2.6-fold higher activity of Cdr1.

We previously reported that Cdr1p localization to the plasma membrane (PM) is critical for its efflux activity^22^, hence we used *CDR1-GFP* tagged strain KS052^23^ to investigate Cdr1p localization after treatment with capric acid or FLC-capric acid combination. Localization of Cdr1-GFP in PM was observed only in *C. albicans* cells with or without capric acid (Fig. 2C). Upon FLC or FLC-capric acid treatment, mislocalization of Cdr1p from PM to the inside of the cells was observed (Fig. 2C).

### Sterol composition is altered in *C. albicans* treated with capric acid or fluconazole-capric acid combination

The presence of ergosterol in *C. albicans*’ cells and the fluidity of the membranes are major factors affecting both localization of Cdr1 in PM as well as its efflux activity^22^. Mislocalization of Cdr1p observed after FLC-capric acid treatment (Fig. 2C) prompted investigation of the content of sterols in the PM, as well as PM fluidity (Table 1).

**Table 1:** Sterols (μg/mg dry mass of isolated PM lipids, means ± SD, *n* = 3) in *C. albicans* CAF2-1 grown for 8 h in the following conditions: control without antimicrobial agents; FLC—treated with fluconazole 4 μg/mL; Capric acid—treated with capric acid 45.3 μg/mL; FLC + Capric acid—simultaneously treated with fluconazole 4 μg/mL and capric acid 45.3 μg/mL. General polarization values (GP; means ± SD, *n* = 6) of Laurdan incorporated into PMs of *C. albicans* CAF2-1 grown for 8 h with antimicrobial agent(s). Statistical analysis was performed by comparing cells treated with antimicrobial agent(s) with the corresponding untreated controls (*p < 0.05; **p < 0.01; ***p < 0.001).

	Control	FLC	Capric acid	FLC + Capric acid
Ergosterol	35.14 ± 2.84	4.53 ± 0.3***	54.39 ± 0.9***	13.47 ± 1.65***
Lanosterol	7.19 ± 1.36	20.96 ± 2.27*	8.77 ± 0.72	14.37 ± 1.19*
GP	−0.26 ± 0.04	−0.14 ± 0.02*	0.13 ± 0.05***	−0.02 ± 0.001**

The PM of *C. albicans* cells treated with FLC had an almost eightfold lower ergosterol content, but a threefold higher concentration of lanosterol (Table 1). Capric acid treatment resulted in a 1.6-fold increase in ergosterol concentration in the PM, with no significant differences in lanosterol content. However, the combination of both compounds leads to 2.6-fold decrease in ergosterol content and twofold increase in lanosterol content, compared with untreated cells. Treatment with both compounds appeared to result in higher Laurdan’s general polarization (GP) values, which indicates more rigid PMs. The highest GP values were observed in cells treated with capric acid alone, which indicated almost fourfold more rigid PM, than in case of untreated cells. The treatment with FLC, or FLC-capric acid combination has led to 2- or threefold more rigid PMs, respectively (Table 1).

### *ERG11* expression is affected differently in *C. albicans* treated with capric acid or fluconazole-capric acid combination

**Figure 3:**
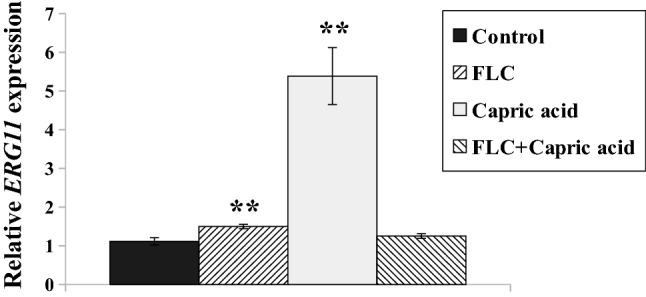
Relative *ERG11* gene expression in the *C. albicans* CAF2-1 grown for 8 h in the following conditions: control without antimicrobial agents, FLC—treated with fluconazole 4 μg/mL; Capric acid—treated with capric acid 45.3 μg/mL; FLC + Capric acid—simultaneously treated with fluconazole 4 μg/mL and capric acid 45.3 μg/mL. Gene expression levels are reported as means ± SD of 2^−ΔΔCT^ values (*n* = 3), normalized to 1 for control conditions. Statistical analysis was performed by comparing the *ERG11* expression level of cells treated with antimicrobial agent(s) with the corresponding untreated control. Statistical significance was presented as follows: **p < 0.01.

Alterations in sterol profiles in FLC-resistant *C. albicans* isolates are mostly the result of over- or underexpression of the *ERG11* gene^4,28^. *ERG11* encodes an enzyme, lanosterol 14α-demethylase, which directly converts lanosterol into further ergosterol metabolites^29^. To understand the variations in sterol content in *C. albicans* treated with capric acid or an FLC–capric acid combination (Table 1), we evaluated *ERG11* gene expression (Fig. 3).

An increase in *ERG11* expression was observed in response to FLC or capric acid treatment (Fig. 3). FLC treatment lead to only 1.5-fold higher *ERG11* gene expression, while capric acid treatment resulted in 5.4-fold higher *ERG11* expression. Combining both compounds reverted the effect and *ERG11* expression resembled that of untreated cells (Fig. 3).

### Ergosterol overproduction induced by treating ***C. albicans*** with capric acid inhibits plasma membrane permeabilization and K^+^ leakage by amphotericin B

Combination treatment with capric acid and AMB lead to an antagonistic effect against *C. albicans* (Fig. 1C). Treatment with capric acid resulted in higher content of ergosterol in PM (Table 1). As *C. albicans* may acquire resistance to AMB by overproduction of ergosterol^13^, we decided to investigate the influence of AMB on *C. albicans* cells grown in the presence of capric acid.

**Figure 4:**
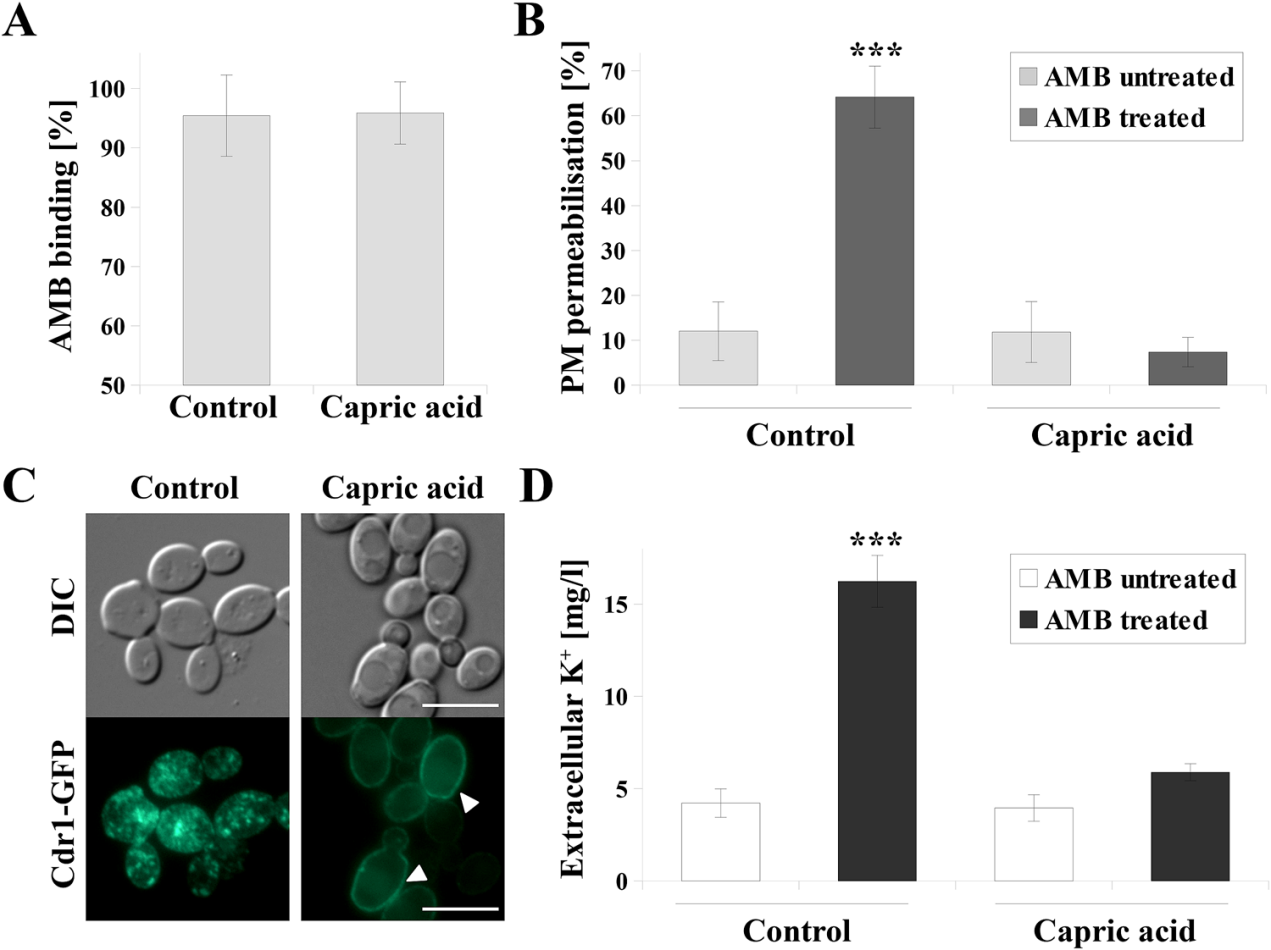
(**A**) Amphotericin B (AMB) binding to *C. albicans* CAF2-1 cells (means ± SD, *n* = 3). (**B**) PM permeabilization measured by counting propidium iodide (PI) positive cells (means ± SD, *n* = 6). (**C**) Fluorescence micrographs of the subcellular localization of the Cdr1-GFP protein in the *C. albicans* KS052 (CAF2-1 *CDR1-GFP*) after treatment with AMB. (**D**) Concentration of extracellular potassium (K^+^) (means ± SD, *n* = 3). For the experiments *C. albicans* was grown for 8 h without capric acid (control) or treated with capric acid 45.3 μg/mL. Following the culture, cells were washed to get rid of capric acid and treated with 2 µg/mL of AMB for 1 h. Statistical analyses (**B** and **D**) were performed by comparing the results cells treated with AMB with the corresponding untreated control. Scale bar = 5 μm. Statistical significance was presented as follows: ***p < 0.001.

The mechanism of AMB toxicity is the binding to fungal ergosterol, permeabilization of PM and cytoplasm leakage^30,31^. Our previous studies indicated that the mechanism of action of AMB also involved delocalization of membrane proteins, including Cdr1p^27^. Here, we characterized the effect of AMB in *C. albicans* CAF2-1 cells treated with capric acid (Fig. 4).

The binding of AMB to *C. albicans* cells (Fig. 4A) was monitored by spectrophotometry at 409 nm^32^. In *C. albicans* CAF2-1 cells treated for 8 h with 45.3 µg/mL of capric acid, AMB binding to *C. albicans* were at a similar level (> 95%) as was observed in untreated cells and no statistically significant difference was observed. However, AMB-treatment of *C. albicans* cells not also treated with capric acid, resulted in permeabilization of PM in ~ 65% of cells (Fig. 4B). In the presence of capric acid, PM was permeabilized in about 10% of AMB-treated cells, which was comparable to untreated cells (Fig. 4B).

*C. albicans* cells grown with AMB and without capric acid featured dispersion of the Cdr1-GFP protein inside the cells and delocalization from the PM (Fig. 4C). When cells were cultured in the presence of capric acid, PM localization of Cdr1-GFP was observed even after AMB treatment (Fig. 4C).

Cytoplasm leakage was evaluated by measuring the extracellular concentration of potassium ions (K^+^), using inductively coupled plasma atomic emission spectroscopy (ICP-AES) (Fig. 4D). *C. albicans* cells grown in the presence of capric acid and treated with AMB were characterized by almost threefold lower extracellular K^+^ concentration than cells not treated with capric acid. Capric acid used alone did not K^+^ efflux from the cells, which was similar to entirely untreated *C. albicans* cells (Fig. 4D).

## Discussion

Contemporary medicine is faced with the challenge of the development of resistance among *Candida* spp. to the common antifungal drug classes including azoles and polyenes. Cytotoxicity of polyenes is another major concern. For these reasons, there is an urgent need for alternative treatment options to the currently available antifungal regimens. Investigating natural compounds in combination with antifungal drugs have recently gained increasing research interest. In this study we report the effect of capric acid on the activities of the most commonly used azole and polyene drugs, FLC and AMB, respectively. Capric acid is a short-chain (C10:0) fatty acid (FA) synthesized by the yeast *S. boulardii* and is commonly used as an anti-diarrheal dietary supplement^18^. Our previous studies indicated that capric acid has anti-adhesion activity and inhibits the adhesion of *C. albicans* cells to abiotic surfaces and intestinal epithelial cells^18^. Furthermore, not only does capric acid display no cytotoxic effects towards human cells^33^, but it has also been reported to inhibit proliferation of different tumor cell lines^34^.

In this study, capric acid was toxic to the *C. albicans ERG11* deletion mutant (*erg11Δ/Δ*) which was as a result negative for ergosterol (Fig. 1A). It has been previously suggested that the presence of ergosterol in fungal cell membranes is crucial for the cellular response to fatty acid treatment^17^. In addition, it has been shown that fungal species lacking sterols are highly sensitive to heptadecenoic acid^35^. Similarly, *S. cerevisiae* cells deficient in ergosterol due to deletion of the *ERG4* gene showed increased sensitivity to undecanoic and lauric acids^36^. The *C. albicans erg11Δ/Δ* strain is also a model example of azole-treated fungal cells^22^, which has been previously used in typing synergistic additives to azole drugs^24^. In our study, the combination of capric acid with FLC was shown to reduce the growth of *C. albicans* CAF2-1 to a greater extent than treatment with FLC alone (Fig. 1B). Similarly, the addition of capric acid enhanced the effect of FLC on clinical, FLC-resistant *C. albicans* isolates. The data was included in Supplementary Information (Fig. S1). In case of *C. albicans* CAF2-1, this difference did not affect the MIC_50_ value of FLC (MIC_50_ = 2 µg/mL), but we found a promising effect of the combination FLC and capric acid on Cdr1p (Fig. 2). Separately, either FLC or capric acid induced expression of *CDR1* gene (Fig. 2A). However, upon combination of both compounds, the expression of *CDR1* reverted to a similar level to that of untreated *C. albicans* cells (Fig. 2A). This effect, as well as Cdr1p mislocalization (Fig. 2C), lead to highly reduced efflux activity of this protein (Fig. 2B). Detection of Cdr1 proteins, which act as inhibitors of *C. albicans*, is one of strategies in overcoming drug resistance to azole drugs^37^. The Cdr1 inhibitors described to date, such as enniatin A and beauvericin, are ionophores which induce the permeability of the cell membrane for ions and display cytotoxic effects towards human cells^38^. This effect is not observed in the case of capric acid, which does not display cytotoxicity towards human cell lines^34^.

Mislocalization of Cdr1p after treatment with FLC-capric acid combination was associated with reduced ergosterol concentration in the PM of *C. albicans* (Table 1). A similar effect was observed in ergosterol-depleted *C. albicans erg11Δ/Δ* strains, in which Cdr1 delocalized into vacuoles^22^. Furthermore, we had previously described that reduced ergosterol levels due to FLC treatment leads to mislocalization of Cdr1^22,23^. In our study, upon either FLC or FLC-capric acid treatment, we observed simultaneous mislocalization of Cdr1p (Fig. 2C) and reduced ergosterol concentration (Table 1). Inhibition of ergosterol biosynthesis most likely occurred at the step of lanosterol demethylation, catalyzed by Erg11p, due to higher content of lanosterol in above mentioned cases (Table 1). Interestingly, in the case of treating *C. albicans* with capric acid alone, we observed high overproduction of ergosterol and rigidification of PM. Such an effect is commonly described in the case of *Candida* spp. strains resistant towards AMB^12^. Therefore, the presence of capric acid was found to almost completely reduce the activity of AMB against *C. albicans* CAF2-1 (Fig. 1C). This is especially important as in clinical practice, apart from looking for additives that would enhance the activity of antifungal drugs, attention is also paid to possible antagonistic interactions. A known example is the interaction of FLC with the anti-cancer drug vincristine, which leads to a neurotoxic effect in patients^21^. The use of FLC during therapy with drugs whose metabolism is mediated by the human cytochrome P-450 is avoided^20^. On the other hand, AMB was reported to display antagonistic effects when administered with immunosuppressants (such as cyclosporin A) or diuretics (such as furosemide)^20^.

Free FAs have been previously reported to directly interact with AMB and inhibit its antifungal activity^39^. Such a relationship has been described so far for myristic, palmitic and stearic acids^39^. To eliminate the effect of a potential direct interaction of capric acid with AMB, *C. albicans* cells were first cultured in the presence of capric acid to an early exponential growth phase of growth and then treated with AMB. It was found that AMB did not cause membrane permeabilization or delocalization of the Cdr1p in *C. albicans* CAF2-1 cells if grown in presence of capric acid (Fig. 4B, C). At this stage of the study, it was concluded that the reduced PM permeabilization occurs despite complete binding of AMB to *C. albicans* cells, regardless if cultured with or without capric acid (Fig. 4A). Additionally, lack of differences in extracellular K^+^ concentration after AMB treatment suggests reduced cytoplasm leakage as a result of AMB treatment. This concludes that, AMB should not be administered among dietary supplements containing capric acid or *S. boulardii* cells.

## Conclusions

Capric acid, produced by *S. boulardii* probiotic yeast, may be a promising additive for use in combination with azole drugs. Capric acid in the combination with FLC has led to reduced efflux activity of Cdr1p as a result of its relocalization from the PM to the interior of the cell. Due to the crucial role of ergosterol in Cdr1p PM localization, the above effect occurred due to inhibitory effect of FLC-capric acid combination of ergosterol biosynthesis. On the other hand, capric acid alone stimulated ergosterol production in *C. albicans*, which in turn generated cross resistance towards AMB and inhibited its toxic mechanisms towards *C. albicans* cells.

## Materials and methods

### Chemicals

Chemicals and reagents were purchased from the following suppliers: capric acid, fluconazole (FLC), conventional amphotericin B (AMB), 2-deoxy-d-glucose, rhodamine 6G (R6G), laurdan, β-mercaptoethanol (BME), ethylenediaminetetraacetic acid (EDTA), cholesterol, and BSTFA/TMCS (N,O-bis(trimethylsilyl) trifluoroacetamide/trimethylchlorosilane) were purchased from Sigma-Aldrich (St. Louis, MO, USA); D-glucose, bacteriological agar, propidium iodide (PI), zymolyase, D-sorbitol were purchased from Bioshop (Ontario, Canada); peptone and yeast extract (YE) were purchased from Becton Dickinson (Franklin Lakes, NJ, USA); chloroform and methanol were purchased from Chempur (Bangalore, India); Total RNA Mini Kit was purchased from A&A Biotechnology (Gdynia, Poland). All chemicals were high purity grade.

### Strains and growth conditions

The *C. albicans* strains used in this study are listed in Table 2. CAF2-1 was a gift from Professor D. Sanglard (Lausanne, Switzerland)^40^. KS028 and KS052 were previously generated by our team^22,23^. Strains were routinely pre-grown at 28 °C on YPD medium (1% YE, 1% peptone, 2% glucose) in a shaking incubator (120 rpm)^22,23^. Agar in a final concentration of 2% was used for medium solidification^22,23^.

**Table 2:** *C. albicans* strains used in the study.

Strain	Genotype	References
CAF2-1	*ura3Δ::imm434/URA3*	Fonzi and Irwin^40^
KS028	*ura3Δ::imm434/URA3* *erg11Δ::SAT1-FLIP/erg11Δ::FRT*	Suchodolski et al.^22^
KS052	*ura3Δ::imm434/URA3* *CDR1/CDR1-GFP-NAT1*	Suchodolski et al.^23^

For most of the experiments, cells were grown in 20 mL of YPD medium, at 28 °C, shaking at 120 rpm with a starting A_600_ = 0.1^22,23^. Cells were incubated with not treatment, or with capric acid, FLC, or the combination of both drugs added at t = 0 h until they reached the early logarithmic phase, typically after 8 h. Cells were then centrifuged at 4,500 rpm for 5 min, washed twice with either phosphate-buffered saline (PBS), 50 mM HEPES–NaOH buffer (pH 7.0) or 0.9% saline, and resuspended in either PBS, HEPES–NaOH or 0.9% saline to the target A_600_.

### Phenotypic tests

Experiments were conducted as previously described^22^. Briefly, CAF2-1 and KS028 strain suspensions (PBS; A_600_ = 0.7; prepared from overnight YPD cultures) were serially diluted up until 10^−3^. Of each dilution, from 10^0^ to 10^−3^, 2 µL were spotted onto YPD agar with or without 22.7, 45.3 and 90.6 µg/mL of capric acid. After cultivation for 48 h at 28 °C, the plates were photographed using a FastGene B/G GelPic imaging box (Nippon Genetics, Tokyo, Japan).

### Determination of growth in the presence of antibiotics

Experiments were performed in compliance with the Clinical and Laboratory Standards Institute (2008), 3rd ed. M27-A3 with described modifications^24^. Briefly, the growth was determined by serially diluting FLC, AMB, or a combination of each of the drug with capric acid in YPD medium using 96-well sterile plates (Sarstedt; Stare Babice, Poland) and then inoculated with CAF2-1 cells at a final A_600_ of 0.01. After incubating at 28 °C for 24 h, A_600_ was measured (ASYS UVM 340, Biogenet). The percentage of growth was determined by normalizing A_600_ in the control experiments (without antimicrobial agents) as 100%.

### Microscopic studies of Cdr1-GFP

The strain KS052, grown in the presence of FLC, capric acid, or combination of both drugs was resuspended in PBS, concentrated and observed under a Zeiss Axio Imager A2 microscope equipped with a Zeiss Axiocam 503 mono microscope camera and a Zeiss HBO100 mercury lamp (Carl Zeiss AG, Oberkochen, Germany).

### R6G efflux assay

Suspensions of CAF2-1, in 25 mL HEPES–NaOH; (A_600_ = 1.0) grown in the presence of FLC, capric acid, or combination of both compounds were treated with 2-deoxy-D-glucose and stained with R6G according to the protocol previously described by Szczepaniak et al.^27^ In each of the conditions, the R6G uptake was estimated to be ≥ 95%. Fluorescence intensities (IFs) were collected 15 min after R6G efflux initiation and normalized to 1 for the efflux activity of untreated CAF2-1 cells.

### Real time polymerase chain reaction

RNA was isolated from the CAF2-1 grown in the presence of FLC, capric acid, or combination of both drugs (PBS; A_600_ = 10) using the Total RNA Mini Kit (A&A Biotechnology). Synthesis of cDNA and calculation of gene expression levels were performed as previously described^41^. The following gene-specific primers were used: ACT1F (5′-TCCAGCTTTCTACGTTTCCA-3′), ACT1R (5′-GTCAAGTCTCTACCAGCCAA-3′), CDR1F (5′-TTTAGCCAGAACTTTCACTCATGATT-3′), CDR1R (5′-TATTTATTTCTTCATGTTCATATGGATTGA-3′), ERG11F (5′-TTTGGTGGTGGTAGACATA-3′), ERG11R (5′-GAACTATAATCAGGGTCAGG-3′).

### Isolation of plasma membranes (PMs) and sterol analysis

PMs were isolated from suspensions of CAF2-1 grown in the presence of FLC, capric acid, or combination of both compounds (PBS; concentrated to A_600_ = 20) according to the previously reported method^23^. Briefly, cells were resuspended in lysis medium (1 M sorbitol, 0.1 M EDTA, 1% BME, 3 mg/mL zymolyase) and incubated at 37 °C for 30 min. Protoplasts were then washed with 1.2 M sorbitol, lysed by ice-cold H_2_O_dd_ shock and disrupted by sonication (5-s cycles for 2 min at 4 °C) using an ultrasonic processor (SONICS Vibra-cell VCX 130). Cell lysate was centrifuged at 10,000 rpm; at 4 °C for 10 min to remove unbroken material, and the supernatant was ultracentrifuged at 100,000 rpm at 4 °C for 60 min using a Micro Ultracentrifuge CS150FNX (Hitachi; Tokyo, Japan). The crude PM pellets were suspended in a chloroform–methanol solution (1:2 v/v). The chloroform layer was concentrated using nitrogen gas after vigorous stirring at 4 °C for 16 h. PM fractions were derivatizated with BSTFA-TMCS and sterol analysis was performed by gas chromatography mass spectrometry (GC–MS) with cholesterol as an internal standard, following previously described protocol^22,23^.

### Membrane fluidity assessment

The membrane fluidity assay was performed according to previously described protocol^22^, with modifications. Briefly, CAF2-1 were incubated for 8 h with 22.7, 45.3 and 90.6 µg/mL of capric acid in YPD. The cells were then centrifuged at 4,500 rpm for 5 min), washed twice with PBS and resuspended in 3 mL PBS (A_600_ = 0.1). Suspensions were labeled with Laurdan at a final concentration of 5 × 10^−6^ M for 20 min at 25 °C in darkness. The probes were excited at 366 nm (Ex slit = 10 nm), and fluorescence spectra were recorded at 400–550 nm (Em slit = 2.5 nm) (PMT voltage = 400 V) using a fluorescence spectrophotometer equipped with a xenon lamp (HITACHI F-4500, Hitachi, Tokyo, Japan). For data analyses, modified general polarization (GP) was calculated as follows: the difference of the sum of IFs from 425 to 450 nm and the sum from 475 to 525 nm, divided by the sum of IFs from 425 to 450 nm and from 475 to 525 nm.

### Assessment of plasma membrane (PM) permeability

To assess the effects of AMB on CAF2-1 PM permeability we followed the PI assay^42^, with modifications. Briefly, suspensions of CAF2-1 grown with or without capric acid (0.9% saline; A_600_ = 1) were exposed to 2 µg/mL AMB for 1 h at 28 °C, pelleted, washed twice with 0.9% saline and resuspended in 0.9% saline with 6 µM PI for 5 min. The cells were then pelleted by centrifugation, washed twice with 0.9% saline, concentrated and observed under a Zeiss Axio Imager A2 microscope equipped with a Zeiss Axiocam 503 mono microscope camera and a Zeiss HBO100 mercury lamp. The percentage of permeabilization was evaluated by counting PI positive cells out of at least 100 cells in each replicate.

### Cytoplasmic potassium ions (K^+^) leakage

To assess the effects of AMB on CAF2-1, cytoplasm leakage suspensions of CAF2-1 grown with or without capric acid (0.9% saline; A_600_ = 1) were exposed to 2 µg/mL AMB for 1 h at 28 °C and pelleted. The supernatants were evaluated for the K^+^ concentrations using inductively coupled plasma atomic emission spectroscopy (ICP-AES).

### Determination of AMB binding to cells

The assay was performed according to Skwarecki et al.^32^, with modifications. Briefly, suspensions of CAF2-1 grown with or without capric acid (0.9% saline; A_600_ = 1) were exposed to 2 µg/mL AMB for 1 h at 28 °C and pelleted. A_409_ of the supernatants was determined using ASYS UVM 340 Biogenet. The percentage of AMB binding was evaluated in accordance with 2 µg/mL AMB in 0.9% saline (100%) and supernatant of AMB untreated cells (0%).

### Statistical analysis

At least three independent replicates were performed for each experiment. Statistical significance was determined using Student’s *t* test (binomial, unpaired).

## Supplementary Information

Supplementary Information 1.
