# Supplementary material for: Capric acid secreted by Saccharomyces boulardii influences the susceptibility of Candida albicans to fluconazole and amphotericin B
Source: Sci Rep. 2021 Mar 22;11:6519. doi: 10.1038/s41598-021-86012-9 (PMC7985486; doi:10.1038/s41598-021-86012-9)
Supplement: Supplementary file 1 — Supplementary Information 1. [file 41598_2021_86012_MOESM1_ESM.docx]

**Supplementary Materials**

**Title:** Capric acid secreted by *Saccharomyces boulardii* influences the susceptibility of *Candida albicans* to fluconazole and amphotericin B

**Authors:** Jakub Suchodolski^1*^, Daria Derkacz^1^, Przemysław Bernat^2^, Anna Krasowska^1^

^1^Department of Biotransformation, Faculty of Biotechnology, University of Wroclaw, 50-383 Wrocław, Joliot-Curie 14A, Poland; jakub.suchodolski@uwr.edu.pl (J.S.); daria.derkacz@uwr.edu.pl (D.D.); anna.krasowska@uwr.edu.pl (A.K.)

^2^Department of Industrial Microbiology and Biotechnology, Faculty of Biology and Environmental Protection, University of Łódź, 90-237 Łódź, Banacha 12/16, Poland; przemyslaw.bernat@biol.uni.lodz.pl (P.B.)

^*^Correspondence: jakub.suchodolski@uwr.edu.pl

**Abstract**

The effect of capric acid, secreted by the probiotic yeasts *Saccharomyces boulardii*, was evaluated on the activities of fluconazole (FLC) and amphotericin B (AMB) against pathogenic *Candida albicans* fungus. The findings indicated that capric acid may be a promising additive for use in combination with FLC. A FLC-capric acid combination led to reduced efflux activity of multidrug resistance (MDR) transporter Cdr1p by causing it to relocalize from the plasma membrane (PM) to the interior of the cell. The above effect occurred due to inhibitory effect of FLC-capric acid combination of ergosterol biosynthesis. However, capric acid alone stimulated ergosterol production in *C. albicans*, which in turn generated cross resistance towards AMB and inhibited its action (PM permeabilization and cytoplasm leakage) against *C. albicans* cells. This concluded that AMB should not be administered among dietary supplements containing capric acid or *S. boulardii* cells.

**Keywords:** *Candida albicans*, *Saccharomyces boulardii*, capric acid (C10:0), ergosterol, amphotericin B, fluconazole.

**Capric acid has a synergistic effect in combination with fluconazole against clinical, fluconazole-resistant *C. albicans* isolates**

The effect of FLC-capric acid combination was evaluated on clinical, FLC-resistant *C. albicans* isolates. *C. albicans* Gu4 and Gu5 strains were isolated from a patient before and after fluconazole administration, respectively. Azole-resistance of Gu5 origins in overexpression of *CDR1* and *CDR2*. *C. albicans* B3 and B4 strains were isolated from a patient before and after fluconazole administration, respectively. Azole-resistance of B4 results from overexpression of *MDR1*.


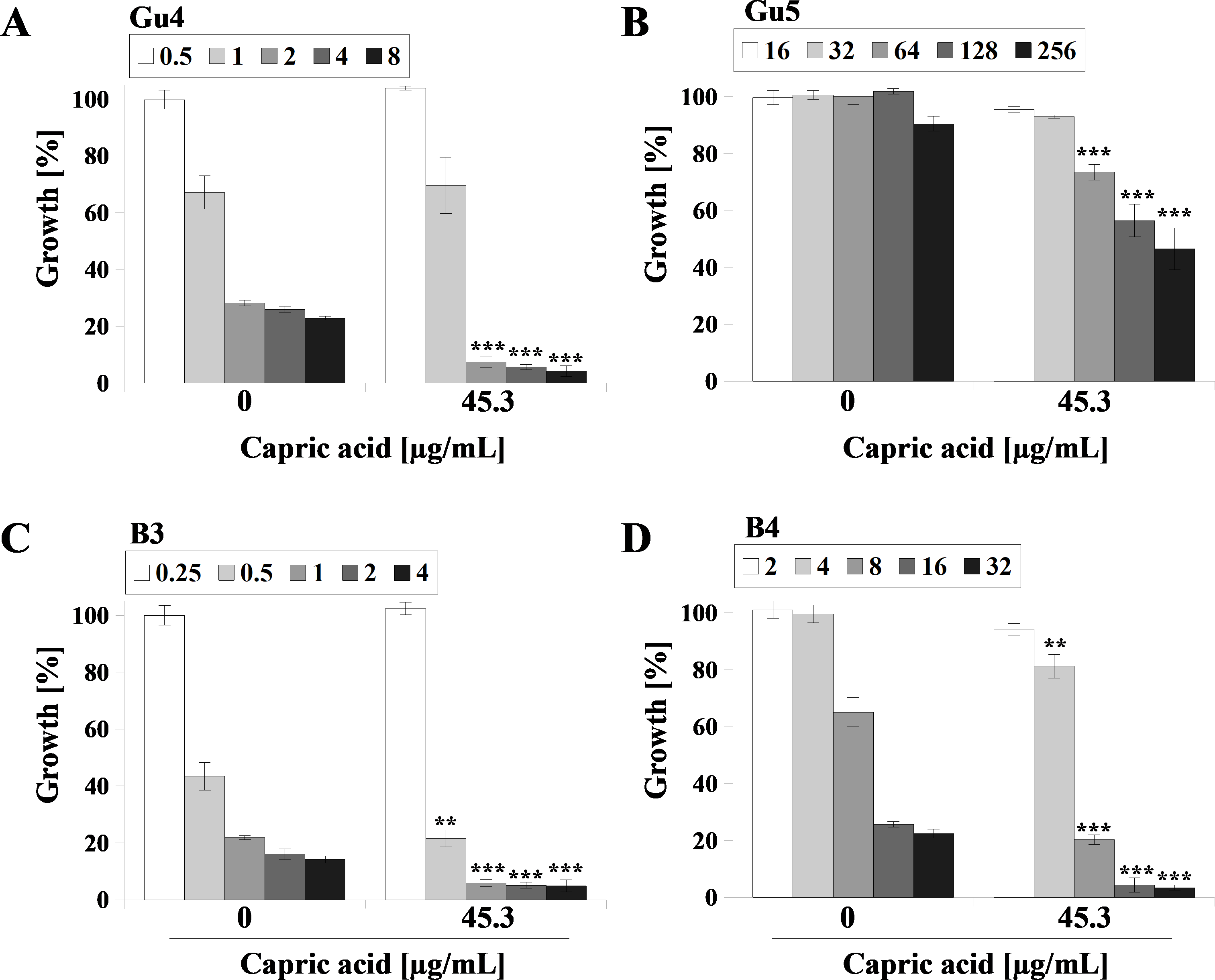


**Figure S1.** Percentage of growth of the *C. albicans* Gu4 (**A**), Gu5 (**B**), B3 (**C**), and B4 (**D**) strains in the presence of fluconazole (μg/mL, chart legend) and in presence of 45.3 μg/mL capric acid (means ± SD, *n* = 3). Statistical significance in all cases is presented as follows: ***p* < 0.01; ****p* < 0.001.

All strains were a generous gifts from prof. S. Milewski (Gdańsk, Poland) and prof. J. Morschhäuser (Wurzburg, Germany). They are originally referenced in: *Franz, R., Ruhnke, M. & Morschhäuser, J. Molecular aspects of fluconazole resistance development in Candida albicans. Mycoses* ***42****, 453–458, (1999).*
